# SELF-PERCEIVED PREPAREDNESS, AWARENESS OF RESOURCES, AND DESIRE TO INSTITUTIONALIZE AMONG CAREGIVERS OF VETERANS

**DOI:** 10.1093/geroni/igae098.1093

**Published:** 2024-12-31

**Authors:** Marianne Desir, Sandra Garcia-Davis, Richard Munoz, Mary Jo Pugh, Ranak Trivedi, Luci Leykum, Stuti Dang

**Affiliations:** Miami Veterans Affairs Healthcare System, Miami, Florida, United States; University of Miami, Miami, Florida, United States; South Florida Veteran Affairs Foundation for Research and Education, Miami, Florida, United States; University of Utah, Salt Lake City, Utah, United States; Stanford University/VA Palo Alto Healthcare System, Palo Alto, California, United States; South Texas Veterans Health Care System, Austin, Texas, United States; Miami VA Healthcare System, Miami, Florida, United States

## Abstract

We analyzed the HERO CARE survey responses of 2,925 caregiver/Veteran dyads to: 1) describe the extent to which caregivers of older Veterans feel prepared for their caregiving tasks; 2) assess whether caregiver awareness of VA caregiver support resources was associated with feelings of preparedness; and 3) identify impact of caregiver feelings of preparedness and odds of caregiver interest in institutionalization of Veterans care recipients. Caregivers described themselves as, “Not at all prepared,” Somewhat prepared,” or “Very prepared” for their caregiving tasks. We calculated descriptive statistics for Veterans and caregivers and compared them by levels of caregiver preparedness using chi-square tests and t-tests. We then conducted adjusted multivariable logistic regression analyses to assess the association between caregiver preparedness and awareness of VA caregiver support services, and between desire to institutionalize and caregiver preparedness. Caregivers were mostly non-Hispanic white (73.3%), female (83.3%), and ≥ 65 years old (72.8%); 62.6% of caregivers felt very prepared, 34.7% felt somewhat prepared and 2.7% felt not at all prepared. Caregivers who were aware of at least one VA caregiver support resource were 33% more likely to feel very prepared compared to caregivers who were not aware of at least one resource (OR: 1.3; 95% CI: 1.1, 1.6; p<0.01). Caregivers who felt very prepared for caregiving were 39% less likely to have desire to institutionalize compared to caregivers who did not feel very prepared (OR: 0.6; 95% CI: 0.5-0.8; p <0.001). Veteran-caregiver dyads may benefit from increased awareness of VA resources, including ones that support caregivers.

